# A Shared System of Representation Governing Quantity Discrimination in Canids

**DOI:** 10.3389/fpsyg.2012.00387

**Published:** 2012-10-08

**Authors:** Joseph M. Baker, Justice Morath, Katrina S. Rodzon, Kerry E. Jordan

**Affiliations:** ^1^Multisensory Cognition Lab, Department of Psychology, Utah State UniversityLogan, UT, USA

**Keywords:** Weber’s law, canid, quantity discrimination

## Abstract

One way to investigate the evolution of cognition is to compare the abilities of phylogenetically related species. The domestic dog (*Canis lupus familiaris*), for example, still shares cognitive abilities with the coyote (*Canis latrans*). Both of these canids possess the ability to make psychophysical “less/more” discriminations of food based on quantity. Like many other species including humans, this ability is mediated by Weber’s Law: discrimination of continuous quantities is dependent on the ratio between the two quantities. As two simultaneously presented quantities of food become more similar, choice of the large or small option becomes random in both dogs and coyotes. It remains unknown, however, whether these closely related species within the same family – one domesticated, and one wild – make such quantitative comparisons with comparable accuracy. Has domestication honed or diminished this quantitative ability? Might different selective and ecological pressures facing coyotes drive them to be more or less able to accurately represent and discriminate food quantity than domesticated dogs? This study is an effort to elucidate this question concerning the evolution of non-verbal quantitative cognition. Here, we tested the quantitative discrimination ability of 16 domesticated dogs. Each animal was given nine trials in which two different quantities of food were simultaneously displayed to them. The domesticated dogs’ performance on this task was then compared directly to the data from 16 coyotes’ performance on this same task reported by Baker et al. (2011). The quantitative discrimination abilities between the two species were strikingly similar. Domesticated dogs demonstrated similar quantitative sensitivity as coyotes, suggesting that domestication may not have significantly altered the psychophysical discrimination abilities of canids. Instead, this study provides further evidence for similar non-verbal quantitative abilities across multiple species.

## Introduction

Recent findings (Baker et al., 2011) add coyotes (*Canis latrans*) to the list of known species capable of making psychophysical discriminations of continuous quantities (e.g., see Brannon and Roitman, 2003; Brannon et al., 2010 for review of other species that share similar abilities). Shared among the discrimination abilities of these species is an adherence to Weber’s Law, which states that the ability to discriminate one continuous quantity from another is mediated by the ratio between the two quantities. As this difference approaches a 1:1 ratio (e.g., as the to-be-compared sets are more similar in quantity/have a larger ratio), discrimination becomes more difficult for non-human animals and humans.

Since such findings are common to a wide range of species and methodological approaches, it has been hypothesized that all species may in fact possess an approximate representation of continuous quantities (e.g., Gibbon, 1977; Gallistel, 1989). In humans, looking time paradigms have revealed approximate representations of numerosity in infants (e.g., Wynn, 1998; Spelke, 2000; Xu and Spelke, 2000; Jordan and Brannon, 2006a; Jordan et al., 2008), while explicit choice paradigms demonstrate maintained adherence to Weber’s Law in these representations throughout childhood and adulthood (e.g., Moyer and Landauer, 1967; Jordan and Brannon, 2006b, 2009; Halberda et al., 2008, 2012; Jordan and Baker, 2011). Variations on such experimental approaches used in humans have demonstrated similar abilities in orangutans (Call, 2000), rhesus macaques (Jordan and Brannon, 2006b,c), chimpanzees (Rumbaugh et al., 1987; Beran, 2004, 2010), and other primates (see Brannon et al., 2010 for review). Similar abilities exist in species ranging from newborn chicks (Rugani et al., 2009), rats (Meck and Church, 1983), dogs (Ward and Smuts, 2007), birds (Pepperberg, 1987; Al Aïn et al., 2009), dolphins (Kilian et al., 2003), raccoons (Davis, 1984), insects (van Hateren et al., 1990), amphibians (Krusche et al., 2010), fish (Gòmez-Laplaza and Gerlai, 2011), elephants (Perdue et al., 2012), and many others (see Brannon and Roitman, 2003; Jordan and Brannon, 2009; Brannon et al., 2010; for review).

Such consistent replication of findings across research groups, designs, and species suggests a highly conserved non-verbal system of representation. Moreover, given the ubiquity of approximate quantitative abilities across species, it is likely that such representations are evolutionarily valuable. However, the biological niche filled by various species may also have honed this ability to different degrees, perhaps depending on each species’ need to discriminate quantity. Purposeful domestication, for example, has been shown to affect various aspects of animals’ behavior. Research comparing foraging behaviors between the “wild type” red jungle fowl (*Gallus gallus*) and the domesticated White Leghorn chicken have identified differences that researchers believe may have arisen as a direct result of domestication (Lindqvist et al., 2009). It is hypothesized that when food supplies across generations become stable as a result of purposeful domestication, an animal’s need to exert effort for high quality and quantity foods is diminished. As a result, the foraging behaviors seen across these two species are markedly altered (Lindqvist et al., 2009).

The domestication process between red jungle fowl and White Leghorn chicken is thought to have occurred over the last 8000 years (Fumihito et al., 1994). Comparatively, domestication between two canids, dogs and wolves, is thought to have occurred over the last 30,000 years (Germonpré et al., 2009). In this time, domesticated and wild canids have experienced significant cognitive divergence. For example, differences between these species of canids in social cognition – namely, differences in animals’ responses to communicative cues from humans – have been shown between domestic dogs and wolves (*Canis lupus*; e.g., Hare et al., 2010). It is possible that similar effects of domestication may have differentially shaped quantitative discrimination abilities between coyotes (*C. latrans*) and domestic dogs (*Canis lupus familiaris*). That is, while the quantitative discrimination capacities of both species adhere to Weber’s Law, the ratio needed to detect the larger food option may differ between the two species. Perhaps, much like the changes in social cognition, domestication brings with it differential abilities to perceive the quantity of or base decisions on the quantity of food options. Alternatively, these two species of canids may show similar abilities for quantitative representation, despite their different ecological niches. The current experiment is an effort to address this question. Direct intra-family comparisons, such as that between coyotes (*C. latrans*) and domestic dogs (*Canis lupus familiaris*) offer a unique look at the effect of domestication on quantitative discrimination abilities.

The procedure used by Baker et al. (2011) to show that coyotes can compare and discriminate different quantities was similar to that of Ward and Smuts (2007), who had previously demonstrated that domestic dogs discriminate quantity and that this ability is mediated by Weber’s Law. In both studies, two different quantities of food were prepared out of the animals’ view, after which both options became visible to the animals as they decided which food option to choose and consume. While Baker et al. (2011) showed that coyotes discriminated between different quantities of food and that these discriminations were mediated by Weber’s Law, they did not directly compare the acuity of this quantitative ability with the acuity of domestic dogs. The current experiment makes this comparison by testing dogs with a similar procedure as was used by Baker et al. (2011) to test coyotes. Comparing these data collected by the same research group, we hypothesize that domesticated canines will show similar quantitative discrimination abilities as coyotes.

To answer such questions, here we replicate the Baker et al. (2011) coyote study in domestic dogs. Next, we subject these new data from domestic dogs to direct comparison with the data from coyotes reported by Baker et al. (2011), in order to identify whether quantitative discrimination abilities differ within divergent members of a single biological family – one of which has been domesticated.

## Materials and Methods

### Facilities and experimental animals

This study was conducted at each animal’s home environment in Cache County, UT, USA. All domestic dogs were tested in an indoor or outdoor open space of approximately 6′ × 6′. All sessions were videotaped for later review. To be eligible to participate in the study, an animal had to show willingness to approach the researcher for food. Sixteen domestic dogs of various breeds met this criterion and participated in this study.

### Food make-up and food preparation

*Pup Peroni*^©^ dog treats were used in the current experiment. Each *Pup Peroni*^©^ stick was cut into eight equal-sized pieces approximately 1/4″ × 1/4″. Since each animal was fed regularly, this was considered a high-value “treat” food given in addition to its daily food intake; therefore, the size of the treat pieces were small in order to prevent satiation before the end of the experimental session.

### Procedure

All animals were tested individually. Each animal experienced eight ratio comparisons, and one olfactory control (1:6 ratio comparison) identical to that used by Baker et al. (2011) to determine whether animals were discriminating quantity based on smell alone, for a total of nine trials per session. Each animal experienced one session of testing. The animal’s owner was present for each trial and kept the animal seated at the beginning of each trial by kneeling behind his/her dog and holding it by its collar. This allowed the animal to remain centered while a food quantity was placed on each side. In order to prevent possible non-verbal cues, the owners were asked to close their eyes during each trial.

To begin each trial, the experimenter sat on the ground approximately 3 feet in front of the animal, while the owner sat behind the dog and held it in place in the manner described above. Once the owner had closed his/her eyes, the researcher obtained the appropriate amount of treats for that trial’s quantitative comparisons and placed one quantity in each hand. The side placement of the large option was pseudo-randomized. The treats were kept in a cloth bag that was easily accessible by the experimenter, yet kept the treats out of the animal’s view. Both quantities were then removed from the bag and placed on the ground covered by the experimenter’s hands; the experimenter then removed her hands simultaneously from the two piles, ensuring they were uncovered at the same time. The treats in each pile were placed close together on the ground so that all pieces could be viewed by the animal and were not obstructed by other pieces piling atop each other. Once the dog had looked at both food options, the experimenter instructed the owner to release it so that it could make its choice. A choice was defined as the animal directly sniffing and/or attempting to retrieve a treat from a pile. Once a food choice was made, the experimenter covered and removed the pile not chosen. Thus, no animal retrieved food from the unchosen pile. An animal was considered to have failed to make a choice if it did not approach the researcher within a minute from the start of a trial.

Each animal received all nine quantitative contrasts used with coyotes by Baker et al. (2011) within a session, which included: 1:4; 1:3; 2:5; 1:2; 2:4; 3:5; 2:3; 3:4; and a 1:6 olfactory control trial. Left-right side presentation of the larger quantity for all ratio comparisons was pseudo-counterbalanced and randomized within and across animals. Order of ratio presentation was also randomized within session; as a result, roughly two-thirds of the animals (68.75%) began testing with small ratio comparisons (1:4, 1:3, 2:5, or 1:2). No animal began testing with the olfactory control trial. On the olfactory control trial, the experimenter placed both food options on the ground but did not reveal them visually to the dog, instead keeping them covered with his/her hands. The owner was instructed to release the dog, and a choice was defined as approaching one of the researcher’s hands within 5′ or less; all dogs actually sniffed the chosen hand as well. Once a choice had been made, the food pile was visually revealed to the animal, and the animal was allowed to eat it. Choosing the large and small food quantities with equal frequency on the olfactory control trial would indicate that the dogs were not discriminating different quantities of food by smell alone.

### Data analysis

We hypothesized that, as found in previous studies such as Ward and Smuts (2007) and Baker et al. (2011), animals would choose the larger quantity of food more often than the smaller quantity. We also predicted that accuracy of choice, or percentage of times that the animal chose the larger quantity over the smaller quantity, would improve as the ratio between food choices decreased. Statistical tests of these hypotheses were conducted using R^®^ (R Core Team, 2012), Prism^®^, and Excel^®^ version 10.

First, binomial tests were used to determine if animals chose the larger quantity more often than the smaller quantity. To test whether animals’ ability to choose the larger quantity of food changed as a function of the ratio between large and small food quantities, binary logistic regression was employed (Agresti, 1996). Finally, to determine whether animals’ choice behavior exhibited scalar variability, linear regression analysis was used. For all parametric statistics, the assumptions of homogeneity of variance and normality were met.

## Results

An alpha level of 0.05 was used for all analyses. Significantly more dogs (12 of 16) chose the larger food quantity more often than the smaller food quantity overall, across all trials (binomial sign test, *p* = 0.027). Table 1 shows the individual animal average performance across all ratios. No significant difference was found between the number of dogs more often choosing the large (7 of 16) vs. the small food quantity in the olfactory control trial (binomial sign test, *p* = 0.175), suggesting that the dogs were not discriminating between quantities of food based on olfactory cues alone.

**Table 1:** **Average percentage of times choosing the large option across all ratios for each individual dog and coyote**.

Animal	Dogs	Coyotes
1	37.5	12.5
2	37.5	37.5
3	50	37.5
4	50	50
5	62.5	50
6	62.5	50
7	62.5	62.5
8	62.5	62.5
9	62.5	62.5
10	62.5	62.5
11	62.5	62.5
12	75	75
13	75	75
14	75	75
15	87.5	75
16	87.5	100
Species average	63.28	59.37

*Coyote data were collected by Baker et al. (2011)*.

In order to identify the influence of ratio on dogs’ food option choice, data were subjected to binary logistic regression. As predicted, this model significantly predicted dogs’ food choice behavior (χ^2^ = 4.21, *p* = 0.04, *Cox and Snell pseudo-R^2^* = 0.03), indicating that the ratio between food option does influence animals’ choice behavior. Moreover, as predicted, linear regression analysis identified significant scalar variability in animals’ choice; variability was significantly lower for small compared to large ratio comparisons [*F*(1, 7) = 7.87, *p* < 0.001, *R^2^* = 0.9].

Similar to Baker et al. (2011) findings in coyotes, we also did not identify any learning effects across trials. Animals that began testing sessions on trials with small ratio (e.g., 1:4, 1:3, 2:5, or 1:2) comparisons succeeded on a similar percentage of small ratio trials compared to animals that began testing sessions on trials with large ratio comparisons [e.g., 2:4; 3:5; 2:3; 3:4; *t*(15) = 0.44, *p* = 0.66]. Similarly, there was no significant difference in the percentage of large ratio trials correct between animals that began testing on small compared to large ratio trials [*t*(15) = 1.19, *p* = 0.24]. A laterality bias (i.e., an animal always chooses the right or left food option) was not observed within any animal.

### Comparison with coyotes

In order to compare the relative quantitative discrimination abilities of dogs and coyotes, the new data collected in dogs reported above were directly compared with the data from coyotes reported by Baker et al. (2011). First, we used an independent sample *t*-test to compare the percentage of trials in which each animal chose the larger food option across species. The results of this comparison indicate that the difference in percentage of trials in which the larger option was chosen between dogs ($\bar{x}=63.28\%$, SD = 14.76%) and coyotes ($\bar{x}=59.38\%$, SD = 20.15%) was not statistically significant, *t*(30) = 0.62, *p* = 0.53.

**Figure 1:**
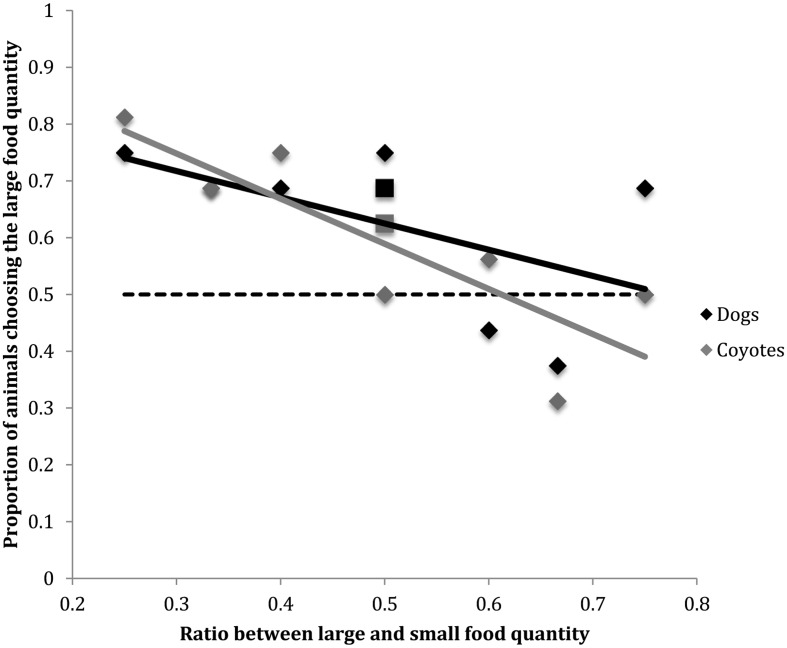
**Proportion of dogs vs. coyotes choosing the larger food quantity as a function of the ratio between large and small food quantities**. Solid lines indicate regression lines (i.e., slopes) for both canid species. Horizontal dashed line indicates chance performance (50%). The negatively sloped regression lines indicate that dog and coyote quantitative choice behavior becomes more random as the ratio between food options approaches 1:1. Coyote data were collected by Baker et al. (2011). The squares displayed at the 0.5 food quantity ratio comparison along the *x*-axis indicate the proportion of animals selecting the larger food quantity when the comparison was 2 vs. 4 food items. The diamonds displayed at the 0.5 food quantity ratio comparison along the *x*-axis indicate the proportion of animals selecting the larger food quantity when the comparison was 1 vs. 2 food items.

Figure 1 shows the proportion of dogs in the current study that chose the larger food quantity as a function of ratio between large and small food choices. Data previously collected in the analogous task by Baker et al. (2011) in coyotes are overlaid onto this figure. The slopes produced by the two species are not significantly different [*F*(1, 13) = 0.48, *p* = 0.49]. The superimposition of the psychophysical functions for these two species suggests that dogs and coyotes may share a similar system of quantity representation.

## Discussion

As predicted, here we show that domestic dogs’ ability to discriminate visual quantities of food items is strikingly similar to their non-domesticated counterparts, the coyote. By using an experimental procedure similar to Baker et al. (2011), we show that domestic dogs’ and coyotes’ quantitative choice behavior adheres to Weber’s Law: discrimination of large vs. small quantities is mediated by the ratio between the two options. Furthermore, quantitative behavior in neither species is mediated by olfactory clues alone. By directly comparing current results in dogs with those of Baker et al. (2011) in coyotes, we find no statistical differences between the two data sets.

Data thus support our hypothesis that the two species exhibit similar quantitative discrimination abilities. These findings further support the claim that certain psychophysical abilities such as ratio-dependent quantitative representation are shared across many species (see Brannon et al., 2010 for review). Results also suggest that such abilities may remain fundamentally unchanged through canid domestication.

While we did not conduct any such analyses here, further tests of possible behavioral differences exhibited by coyotes compared to domestic dogs while foraging could theoretically still reveal differences in quantitative foraging behavior. For example, it remains unknown how testing in the presence of another animal (e.g., competitor or subordinate) may affect quantitative choices in the two species. It is also unknown if there are canid species differences in willingness to exert extended effort due to depletion in food quantity, such as was found between bumblebees (*Bombus impatiens*) and honeybees (*Apis mellifera*; Townsend-Mehler et al., 2011). In this study, bumblebees were willing to change foraging strategy and travel far distances in response to food depletion, though honeybees were not. Thus, there may also be differences in the ways in which species “weight” different quantitative variables in their foraging behavior. Further, it is unknown whether coyotes’ abilities to remember various quantitative comparisons when they are not visible (e.g., in working memory) are comparable to those of dogs found in such studies as Ward and Smuts (2007). It remains possible that the domestication process affected performance on such other quantitative tasks. However, comparative tests of psychophysical discriminations such as those reported here suggest that such abilities may be stable across domestication. Moreover, the ubiquitous nature of such abilities across species further supports the idea of a shared system for non-verbal magnitude representation across many non-human and human organisms.

In addition to providing a comparison to the coyotes tested in Baker et al. (2011), current results provide an approximate replication of Ward and Smuts (2007). There were some small but nevertheless important procedural differences between Ward and Smuts (2007) and the results reported here; for example, the dogs used in Ward and Smuts (2007) were tested in a single testing room environment that was devoid of distractions, while the dogs used in the current study were tested at their own homes. Importantly, despite such environmental differences, similar results were obtained; this contrasts with other recent tests of canine choice behavior in lab vs. open-air environments, where differences in dogs’ choice behavior based on referential emotions displayed by the experimenter have specifically been found (Buttelmann and Tomasello, 2012; though breed also was confounded with testing environment in these conditions). Thus, our replication will do well to justify future research endeavors, which intend to test canid quantitative discrimination abilities in a naturalistic environment. Unfortunately, such replication studies are not currently common in comparative psychology, yet they can provide useful convergent evidence (e.g., Agrillo and Petrazzini, 2012; Irie and Hasegawa, 2012; Perdue et al., 2012).

Our results, however, are not without limitations. For example, because stimulus size and number were positively correlated in the current design, we are unable to discern potential numerical discrimination abilities from potential size/surface area discrimination abilities. Such issues have confounded similar studies of relative quantity judgments in other species of the carnivore order as well (e.g., South American sea lions, Abramson et al., 2011). Future studies on comparative numerical cognition in carnivores, perhaps using a non-food stimulus, should further address this issue by removing the correlation between number and size, as has recently been accomplished in American black bears (Vonk and Beran, 2012). Furthermore, future studies could include a greater number of trials per ratio comparison, enabling for example assessments of functions for individual animals. Because each animal in the current study was only presented with each quantitative contrast once, we were not able to determine consistency of individual choice behavior within each ratio comparison. In the future, it will also be necessary to compare domesticated and non-domesticated canids on their abilities to discriminate quantities that are not both visible sets available at the time of the decision. For example, animals could be tested with items or sets presented sequentially into opaque containers or covered out of sight, so that they would have to hold the quantities perceived in memory and make a final comparison (e.g., Beran et al., 2008; Evans et al., 2009). Such a test may perhaps be more relevant to certain species than others based on their ecology.

Finally, there was in this experiment the potential for experimenters to unintentionally cue the dogs, as the experimenter watched the dogs approach and knew which choice was the larger food quantity. Current data suggest that dogs were not using any such unintentional cues, especially data from the olfactory control trial. On this trial, the dogs’ performance was not above chance, even though this was a very easy comparison (1 vs. 6). Similarly, the dogs did not reach 100% accuracy on all visual trials – even though the experimenter always knew which side held the larger food item – suggesting lack of use of any unintentional human cues. Moreover, as reported by Buttelmann and Tomasello (2012), domestic dogs do not modify their choice behavior when humans display non-meaningful emotional expressions in the direction of one of two choices. The experimenter in the current study remained emotionally neutral throughout all trials. Nevertheless, in the future a more “blind” protocol to make sure the experimenter is not signaling or otherwise cuing the dogs to which option contained the larger amount of food would be useful.

In conclusion, our results demonstrate that domestic dogs possess similar abilities to discriminate visual quantities of food items as non-domesticated coyotes. These results suggest that domestication may not significantly affect quantitative discrimination of visually presented food items. Future studies are needed to further elucidate such issues and to investigate performance across related species in discriminating and basing decisions on other quantitative properties such as number, space, and time. Because such abilities have been shown to exist across many species, it would be interesting to identify whether they, too, maintain relative consistency across the domestication process or whether they are altered in a way not observed here. Consistency across such related species may support our claim that ratio-dependent, approximate quantitative abilities may be shared across many species.

## Conflict of Interest Statement

The authors declare that the research was conducted in the absence of any commercial or financial relationships that could be construed as a potential conflict of interest.
